# Exploring Urological Experience in the COVID-19 Outbreak: American Confederation of Urology (CAU) Survey

**DOI:** 10.1590/S1677-5538.IBJU.2020.S119

**Published:** 2020-07-27

**Authors:** Ana María Autrán-Gómez, Ignacio Tobia, Ricardo Castillejos Molina, Francisco Rodríguez Covarrubias, Frank Benzing, Serena Maruccia, Leonardo de O. Reis, Ramón Rodríguez Lay, Marcelo Torrico De la Reza, Felix Santaella Torres, Andrés Hernández Porras, Alejandro Rodríguez

**Affiliations:** 1 University Hospital Fundación Jiménez Díaz Department of Urology Madrid Spain Department of Urology, University Hospital Fundación Jiménez Díaz, Madrid, Spain; 2 Hospital Italiano de Buenos Aires Department of Urology Buenos Aires Argentina Department of Urology, Hospital Italiano de Buenos Aires, Buenos Aires, Argentina; 3 Instituto Nacional de Ciencias Médicas y Nutrición Salvador Zubirán Department of Urology Mexico City Mexico Department of Urology, Instituto Nacional de Ciencias Médicas y Nutrición Salvador Zubirán, Mexico City, Mexico; 4 Brandenburg Medical School University Hospital Brandenburg der Havel Department of Urology Germany Department of Urology, Brandenburg Medical School University Hospital Brandenburg der Havel, Germany; 5 Institute Zucchi Department of Urology Monza Italy Department of Urology, Institute Zucchi, Monza, Italy; 6 Universidade Estadual de Campinas - Unicamp Departamento de Urologia CampinasSP Brasil Departamento de Urologia, UroScience, Universidade Estadual de Campinas - Unicamp, Campinas, SP, Brasil; 7 Complejo Hospitalario Metropolitano Clinic Urology Service Madrid Panama Clinic Urology Service, Complejo Hospitalario Metropolitano, Caja de Seguro Social Dr. Arnulfo Arias Madrid, Panama; 8 San Simón Higher University Cochabamba Clinic Urology Service Bolivia Clinic Urology Service, Los Olivos, San Simón Higher University Cochabamba, Bolivia; 9 Hospital de Especialidades - Instituto Mexicano del Seguro Social Mexico Centro Médico Nacional Department of Urology Mexico-City Mexico Department of Urology, Centro Médico Nacional, La Raza, Hospital de Especialidades - Instituto Mexicano del Seguro Social Mexico, Mexico-City; 10 Hospital Angeles Tiujana Department of Urology Baja California Norte Mexico Department of Urology, Hospital Angeles Tiujana, Baja California Norte, Mexico; 11 Rochester General Hospital Department of Urology Rochester New York US Department of Urology, Rochester General Hospital, Rochester New York, US

**Keywords:** Surgical Procedures, Operative, COVID-19 diagnostic testing [Supplementary Concept], Outpatients

## Abstract

**Purpose:**

To explore the current situation faced by Latin American urology departments during the COVID-19 Outbreak in terms of knowledge, actions, prioritization of urology practices, and implementation of internal clinical management protocols for inpatients and outpatients.

**Material and Methods:**

A non-validated, structured, self-administered, electronic survey with 35 closed multiple choice questions was conducted in Spanish, Portuguese, Italian, and English and Deutsch versions from April 1st to April 30th, 2020. The survey was distributed through social networks and the official American Confederation of Urology (CAU) website. It was anonymous, mainly addressed to Latin American urologists and urology residents. It included 35 questions exploring different aspects: 1) Personal Protective Equipment (PPE) and internal management protocols for healthcare providers; 2) Priority surgeries and urological urgencies and 3) Inpatient and outpatient care.

**Results:**

Of 864 surveys received, 846 had at least 70% valid responses and were included in the statistical analyses. Surveys corresponded to South America in 62% of the cases, Central America and North America in 29.7%. 12.7% were residents. Regarding to PPE and internal management protocols, 88% confirmed the implementation of specific protocols and 45.4% have not received training to perform a safe clinical practice; only 2.3% reported being infected with COVID-19. 60.9% attended urgent surgeries. The following major uro-oncologic surgeries were reported as high priority: Radical Nephrectomy (RN) 58.4%, and Radical Cystectomy (RC) 57.3%. When we associate the capacity of hospitalization (urologic beds available) and percentage of high-priority surgery performed, we observed that centers with fewer urological beds (10-20) compared to centers with more urological beds (31-40) performed more frequently major urologic cancer surgeries: RN 54.5% vs 60.8% (p=0.0003), RC 53.1% vs 64.9% (p=0.005) respectively.

**Conclusions:**

At the time of writing (May 13th 2020) our data represents a snapshot of COVID-19 outbreak in Latin American urological practices. Our findings have practical implications and should be contextualized considering many factors related to patients and urological care: The variability of health care scenarios, institutional capacity, heterogeneity and burden of urologic disease, impact of surgical indications and decision making when prioritizing and scheduling surgeries in times of COVID-19 pandemic.

## INTRODUCTION

Coronavirus disease 2019 (COVID-19) is an emerging, highly infectious respiratory disease, that is caused by a novel coronavirus, now designated as SARS-CoV-2 (Severe Acute Respiratory Syndrome Coronavirus 2) that was first reported on 31st December 2019 in Wuhan, China and is considered responsible for a cluster of new cases of interstitial pneumonia (1). In response to this serious situation, the World Health Organization (WHO) declared it as a “Global Pandemic” on March 11th, 2020 and called for collaborative efforts of all countries to prevent the rapid spread of COVID-19 (2).

At the time of writing (13th May 2020) 4,262,799 cases were confirmed, 291,981 deaths reported with 187 countries around the world facing this health emergency (3). Many hospitals and urology departments have adapted resources, limited surgeries and delayed diagnostic procedures. Patients scheduled for major elective surgeries have undergone a triage to prioritize oncologic disease, given that delaying treatment may affect oncological patients' survival and quality of life of oncologic patients. Internal management protocols and recommendations for inpatients and outpatients established by different international societies of urology, have been published to optimize patient care (4–6). Many questions regarding short and long-term effects of the pandemic araise: How should urologists act? Have we properly selected patients? Further concerns must be analyzed including different scenarios, health care systems, education, training and health situation with respect to the pandemic at this time.

Nevertheless, the battle against COVID-19 is still continuing worldwide. For Latin-America this threat represents a risk of collapse to all health care systems. The Latin American urological community, is represented by the American Confederation of Urology (CAU) which involves 24 urological societies in 22, conveys different scenarios in professional development and heterogeneous public health care infrastructures and policies influencing the way of facing the pandemic.

## OBJECTIVES

To explore the current situation faced by Latin American urology departments during the COVID-19 emergency in terms of knowledge, actions, prioritization of practices, and implementation of internal clinical management protocols for inpatients and outpatients.

## MATERIAL AND METHODS

This was an electronic survey based on a non-validated, structured, self-administrated questionnaire consisting of 35 closed multiple choice queries. It was conducted in Spanish, Portuguese, Italian and English versions. The survey was opened on April 1st and closed in April 30th 2020. The survey was distributed on-line through social networks, the official CAU website (7) and CAU mailing distribution list available at. <http://www.caunet.org/en/urology>, and anonymous mainly for from Latin American urologists and urology residents. The 35 queries explored different issues: 1) Personal Protective Equipment (PPE) availability and design of internal managment protocols for healthcare staff; 2) Prioritization of surgeries and urological urgencies. The level of priority groups for procedures was categorizing into: a) High priority; b) Low Priority and c) No priority and 3) Inpatient and outpatient care activity.

### Statistical Analysis

Categorical responses were expressed as its absolute values and percentages (%). In each issue analyzed we included if responses were categorized as excluded answers. Descriptive analyses were carried out. All variables were compared using chi-square test and multiple comparisons adjusted by Bonferroni's method. In all cases a p value less than 0.05 was considered as statistically significant. Data analysis was performed using SPSS (Version 22, IBM Corp, New York, U.S.A.).

## RESULTS

Of 864 surveys received, 846 had at least 70% valid responses and were included in the statistical analyses. Surveys corresponded to South America in 62% of the cases (distribution of 522 questionnaires: Argentina 116, Peru 75, Bolivia 70, Ecuador 64, Chile 48, Colombia 48, Venezuela 43, Uruguay 27, Paraguay 18 and Brazil 13), Central America and North America in 29.7% (distribution of 250 questionnaires: Mexico 97, Dominican Republic 34, Panama 26, Guatemala 23, Nicaragua 16, Costa Rica 11, El Salvador 11, Honduras 10, Puerto Rico 10, USA 9 and Cuba 3) and Europe and other countries the remaining 8.3% (distribution of 70 questionnaires: Spain 61, Italy 6, France 1, Portugal 1, Qatar 1, China 1).

Participants' age distribution was: less than 40 years in 36.3%, between 40 and 55 years in 39.5% and older than 55 years in 23.3%. In terms of gender 17% were female. Of 841 surveys which specifing educational level, 12.7% were residents in training; 362 (41.9%) trained urologists reported not having residents under their charge. In terms of urological clinical practice: 70% practiced general urology, 25% uro-oncology and 26% endourology. In terms of practice setting: 363 (43.2%) were in university hospitals and 249 (39.7%) in public and non-academic hospitals. Overall characteristics of the study population are shown in (Table-1).

**Table 1:** General Characteristics of study population.

Characteristics		Number (%)	Number (survey response type)
**Country COVID-19 cases**			**841 (exclusive)**
	<500	239 (28.4)	
	500-1000	119 (14.1)	
	1001-5000	27 (32)	
	> 5000	213 (25.3)	
**Urologic Subspecialty**			**838 (non-exclusive)**
	General Urology	589 (70.3)	
	Uro-oncology	210 (25.1)	
	Andrology	50 (6)	
	Endourology	221 (26.4)	
	Functional Urology	72 (8.6)	
	Pediatric Urology	68 (8.1)	
**Practice Setting**			**841 (non-exclusive)**
	University Hospital	363(43.2)	
	Private Center	461 (55)	
	Public Hospital, non-university	249 (39.7)	
	Military Hospital	49 (12.8)	
	Others	20 (2.4)	
**Urologic Beds**			**810 (exclusive)**
	10-20	582 (71.9)	
	21-30	130 (16)	
	31-40	98 (12.1)	
	More than 40	0	
**Urologic COVID19+Beds**			**777 (exclusive)**
	No cases	646 (83.1)	
	1-5 cases	68 (8.8)	
	6-20 cases	51 (6.6)	
	More than 21 cases	12 (1.5)	

### PPE and internal management protocols

Regarding PPE and internal management protocols for healthcare provides, of 833 valid responses, 733 (88%) confirmed the implementation of specific protocols. Strikingly, only 455 (54.6%) professionals, received training on COVID-19 self-care protection protocols and 45.4% did not receive training to perform a safe clinical practice. Only 2.3% reported being infected with SARS-CoV-2; in contrast, the remaining 87.6% of non-infected urologists reported working in health care centers with proper internal management protocols. Lastly, 91.7% of urologists kept themselves updated about the latest news published publications regarding management protocols.

### Priority of surgeries and urological urgencies

Analyzing surgical activity, 60.9% performed urgent procedures as ureter stentings (double J-stents, pigtail stents) or nephrostomy tube placement due to infection, obstructive lithiasis or both; in 50% of these cases, there was not any COVID-19 protocol available for urological urgencies; in 37.4% of the cases the patient was assumed to be COVID-19 positive without prior testing; and only 13% of patients received COVID-19 testing before hand.

Uro-oncologic surgeries were reported by 43.9% of participants, followed by endourologic procedures in 18.5% (mostly renal colic)(Table-2). Of 777 (100%) surveys completed, the following oncologic surgeries were registered as High-Priority: Orchiectomy 69.3%, Transurethral Resection of Bladder Tumor (TURB) 63.6%, RN 58.4%, RC 57.3% and Radical Prostatectomy 32.4%. In contrast, oncologic surgery was more commonly rendered as Low Priority in Partial Nephrectomy in 54.8% and no oncological surgeries, No Priority: Transurethral Resection of Prostate (TURP) in 48%. Regarding to surgical access: 73.8% of surgeries were performed by open approach and 26.2% by minimally-invasive surgery approach. Laparoscopic or Robotic, RN and Radical Prostatectomy were more frequently major uro-oncologic surgeries. In 43.2% scheduling and prioritizing surgeries as well as decision making was performed by the urologist in charge. 57.7% decided to postpone the surgery when the use of blood derivates was being planned and 42.3% were performed under standard protocol (Table-3). In terms of potential use of Intensive care unit (ICUs) for high-priority surgeries, 75.6% decided to postpone procedures and 13.8% performed them under a COVID-19 internal management protocol. When we associate the capacity of hospitalization (urologic beds available) vs % of the high priority surgeries performed, we observed that centers with fewer urological beds (10-20) vs. those with more urological beds (31-40), the later performed more major urologic cancer surgeries, such as RN (54.5% vs. 60.8; p=0.0003), RC (53.1 vs. 64.9; p=0.005), compared to those with more urological beds (31-40),respectively. Other oncological surgeries such as: Orchiectomy (68.2% vs. 67.7%; p=0.078), TURB (61.7% vs. 62.5%; p=0.110), Penectomy (56.1% vs. 51.1%; p=0.223) and Radical Prostatectomy (30.1% vs. 33.3%; p=0.121) did not show statistically significant differences. On the other hand, 16.9% of urological beds were assigned to COVID-19 positive patients without urological conditions (Table-4).

**Table 2:** Urological activity during COVID-19 period.

Issue		Number (%)	Number (survey response type)
**Types of scheduled surgeries**			**816 (non-exclusive)**
	Only urgencies	497 (60.9)	
	Uro-oncology	358 (43.9)	
	Andrology	12 (1.5)	
	Endourology	151 (18.5)	
	Functional Urology	33 (4)	
	Pediatric Urology	15 (1.8)	
**External consultation management**			**820 (non-exclusive)**
	Closed	405(49.4)	
	Telephone calls	264 (32.2)	
	Teleconsultation (Video Calls: Skype, Facetime, Zoom)	131 (16)	
	No changes	63(7.7)	
	Only follow up visits	187 (22.8)	
**Procedure to follow in urgencies surgeries**			**812 (exclusive)**
	No specific protocol	402 (49.5)	
	Patient is assumed to be COVID-19 Positive Protocol	304 (37.4)	
	Test COVID-19 to evaluate patient status	106 (13.1)	
**Surgical treatment decision maker**			**777 (non-exclusive)**
	Local Uro-oncologic Committee	194 (24.9)	
	Responsible Urologist	336 (43.2)	
	Service Chief	269 (34.6)	
	Uro-oncology Unit Chief	68 (8.8)	

**Table 3:** Priority of urologic surgeries during COVID-19 pandemic in Latin American urologic departments.

Surgery	No priority (%)	Low priority (%)	High priority (%)	Surveys included
Radical prostatectomy	120 (15.8)	394 (51.8)	246 (32.4)	760
Partial nephrectomy	116 (15.6)	408 (54.8)	220 (29.6)	744
Radical nephrectomy	66 (8.6)	252 (33)	446 (58.4)	764
Radical cystectomy	90 (12.1)	228 (30.6)	428 (57.3)	746
TURB	59 (7.7)	221 (28.7)	490 (63.6)	770
Retroperitoneal Lymphadenectomy	118 (16.1)	386 (52.7)	228 (31.1)	732
Orchiectomy	59 (7.7)	177 (23)	534 (69.3)	770
Penectomy	76 (10.1)	251 (33.4)	425 (56.5)	752
BPH	363 (48)	352 (46.6)	41 (5.4)	756
Lithiasis	139 (18.1)	402 (52.2)	229 (29.7)	770

**Table 4:** Association between Capacity of Hospitalization (urologic beds available) vs High Priority Surgeries Performed in COVID-19 pandemic.

	Urologic Beds Number			
Surgery	10-20	21-30	31-40	*p* value
Radical Prostatectomy	157 (30.1)	50 (40)	32 (33.3)	0.121
Partial Nephrectomy	146 (28.6)	39 (31.4)	29 (30.9)	0.286
Radical Nephrectomy	285 (54.5)	93 (74.4)	59 (60.8)	**0.0003**
Radical Cistectomy	270 (53.1)	88 (70.4)	61 (64.9)	**0.005**
TURB	324 (61.7)	91 (71.7)	60 (62.5)	0.110
Retroperitoneal Lymphadenectomy	144 (29)	44 (35.8)	29 (30.9)	0.505
Orchiectomy	362 (68.2)	97 (77)	63 (67.7)	0.078
Penectomy	290 (56.1)	77 (62.6)	47 (51.1)	0.223
BPH surgery	28 (5.4)	8 (6.5)	3 (3.2)	0.556
Lithiasis treatment	157 (29.7)	37 (28.9)	26 (27.1)	0.975

### Inpatient and outpatient care activity

In terms of urologic cancer care, 76.2% reported that their centers continued to provide oncological treatments, including intravesical instillations or chemotherapy, and 25.4% of internal radio-oncology departments continued with a regular treatment schedulea.

Regarding to urologic outpatient's follow-up, many hospitals or healthcare systems have reported the implementating technologic resources into the provision of urologic consultations in order to supply recommendations and prescriptions. Of these, 32.2% used telephone calls and 16% adopted telemedicine thus connecting to platforms as facetime, skype and zoom.

## DISCUSSION

The World Health Organization declared COVID-19 as a “Global pandemic” and public health emergency on March 11th, 2020, calling for collaborative efforts from all countries to prevent the rapid spread of the disease (2). At this time (May 13th, 2020) 4,262,799 cases have been confirmed, 291,981 deaths have been reported with 187 countries around the world are facing this health emergency, which representing a risk of collapse for all health care systems (3).

Many hospitals and healthcare urologic centers around the world have adapted resources, limited surgeries as well as diagnostic procedures and have postponed major elective surgeries. Internal management protocols and recommendations for inpatient and outpatient care, have been provided by different international societies of urology to optimize patients' management, including decreasing the general inflow of patients to hospitals and reducing the number of medical and surgical procedures, therefore ensuring that only urgent and non-deferrable oncological surgeries are performed (4–6).

On February 28th, the president of the Robert-Koch-Institute (RKI) in Berlin, Germany suggested to defer all non-urgent surgeries (8). Similarly, in most of European National Health Systems a reduction of surgical activity was recommended. Several definitions of deferrable and non-deferrable procedures have been proposed by panels of experts from all around the world, taking into account several factors, including the aggressiveness/severity of each disease, the impact of short term delays to care and the availability of alternative treatment modalities (4–6).

Our study provides data from 22 Latin American countries that may contextualize the ongoing recommendations on selection of high-priority major uro-oncologic surgeries as RN 58.4% and RC 57.3% which are more frequently performed in Latin American urologic centers with less capacity of hospitalization (10-20 urologic beds available) compared tocenters with more capacity (31-40 urologic beds available). Our findings have practical implications and should be analysed considering many factors related to patients and urologic care: the variability of health care scenarios, the volume capacity at each center, the volume and variability of urologic disease, the impact of surgical indications and decision making when prioritizing and scheduling surgeries in times of the COVID-19 pandemic. Oderda et al. (9) conducted a survey involving 57 European urological referral centers. They showed that the management of the main urological cancers has been altered dramatically by the COVID-19 pandemic, with most European centers (82%) declaring to be “much” or “very much” affected. Uro-oncological consultations for newly diagnosed cancers and follow-up were more than halved or almost suspended, in 55% and 71% of centers, respectively.

At present, the constant requirement of beds and mechanical ventilators in ICUs has increased due to the influx of critical patients requiring ventilatory support, transforming surgical areas into intensive care spaces, thus decreasing the capacity of surgical areas; making clear that prioritizing urological urgencies is essential. Stensland et al. (10) defined a list of urological conditions and surgical procedures that patients may undergo during the pandemic, stressing a more conservative approach whenever feasible. For example, benign prostate hyperplasia (BPH) and urinary tract stones should be treated only if complications occurs, with catheterization, and nephrostomy or ureteral stenting respectively. Surgery should be maintained just for urological urgencies, such as testicular torsion, refractory gross hematuria or oncologic disease.

Our data reported that 60.9% performed urgent procedurs such as ureteralstenting or nephrostomy placement due to infection, lithiasis or combination of both.

At this time, an adequate use of PPEs for healthcare providers and specific internal management protocols are essential to contain the spread of the virus (11). In this study, 88% of the participants confirmed the implementation of specific protocols in their urologic centers, but only 2.3%, reported being infected with COVID-19. Probably this low percentage of contagion may be due to the period (April 1st - April 30th 2020) in which the survey was conducted; at that time in Latin American countries the number of COVID-19 positive cases reported was lower than in Europe. It is important to emphasize that the number of healthcare providers infected reported around the world is correlated to adequate use and availability of PPE as well as the number of tests performed to confirm the presence of SARS-CoV-2.

During this exceptional situation, most hospitals and healthcare providers in critically affected areas are changing their on-site activity to telehealth medicine in order to reduce hospital visits to the minimum necessary (12, 13). In this context, telemedicine, particularly video consultations have been promoted for reduce the risk of transmission and to facilitate the follow-up in urologic consultations, medical recommendations, prescriptions and the surgical follow-up of discharged. In our data 16% of urologists have implemented the use of telemedicine in order to continue with clinic activity at home.

This survey has several limitations: the participation from some countries was limited, many urologist may not have taken part in the survey due to the number of other surveys exploring the impact of COVIID-19 in urologic practice. To properly interpret our results it is fundamental to consider the variability of health care scenarios across Latin American countries, the hospitalization capacity at each center (beds, mechanical ventilators, ICUs, equipment), the volume and variability of urologic disease, the health situation of each Latin American country with respect to the pandemic at the time of the survey analysis, the impact of surgical indications and decision making when prioritizing as well as scheduling surgeries in times of COVID-19 pandemic.

## CONCLUSIONS

At the time of writing, our data represents a snapshot of COVID-19 outbreak in the Latin American urological practice. Our findings have practical implications and should be contextualized considering many factors related to patients and urological care: the variability of health care scenarios, the volume capacity at each center, the volume and variability of urological disease, the impact of surgical indications and decision making when prioritizing as well as scheduling surgeries in times of COVID-19 pandemic.

The COVID-19 era represents one of the biggest challenges is modern health care history. Urological practice has been severely impaired beyond the tragic effects of this emergency. However, several opportunities for improving urological research, clinical and surgical care of outpatient and inpatient settings have been rapidly developed, creating an excellent feedback of knowledge among the urological community around the world.
